# Cervical Dystonia: From Pathophysiology to Pharmacotherapy

**DOI:** 10.3233/BEN-2012-120270

**Published:** 2013

**Authors:** Sejal Patel, Davide Martino

**Affiliations:** ^1^The Michael Trimble Neuropsychiatry Research GroupDepartment of NeuropsychiatryBSMHFT and University of BirminghamBirminghamUK; ^2^Neuroscience and Trauma CentreBarts and The London School of Medicine and DentistryQueen Mary University LondonLondonUK; ^3^Neurology DepartmentPrincess Royal University HospitalSouth London NHS TrustOrpingtonKentUK

**Keywords:** Botulinum toxin A, botulinum toxin B, cervical dystonia, torticollis, trihexyphenidyl, pharmacotherapy

## Abstract

*Background:* Dystonia is a chronic disorder characterised by an aberration in the control of movement. Sustained co-contraction of opposing agonist and antagonist muscles can cause repetitive and twisting movements, or abnormal postures. Cervical dystonia (CD), often referred to as spasmodic torticollis, is a type of focal dystonia involving the muscles of the neck and sometimes the shoulders.

*Methods:* This systematic review collates the available evidence regarding the safety and efficacy of a range of treatments for CD, focusing on their effectiveness as shown by double-blinded, randomised controlled trials.

*Results:* Our review suggests that botulinum toxin type A (BTA), botulinum toxin type B (BTB) and trihexyphenidyl are safe and efficacious treatments for CD. Evidence shows that botulinum toxin therapies are more reliable for symptomatic relief and have fewer adverse effects than trihexyphenidyl. When comparing BTA to BTB, both are found to have similar clinical benefits, with BTA possibly having a longer duration of action and a marginally better side effect profile. BTB is also safe and probably just as efficacious a treatment in those patients who are unresponsive or have become resistant to BTA.

*Discussion:* The current evidence shows that the pharmacological management of CD relies on BTA and BTB, two agents with established efficacy and tolerability profiles.

## 1. Introduction

Dystonia is a chronic disorder characterised by an aberration in the control of movement. Sustained co-contraction of opposing agonist and antagonist muscles can cause repetitive and twisting movements, or abnormal posture [1].

Cervical dystonia (CD), often referred to as spasmodic torticollis, is a focal subset of dystonia involving the muscles of the neck and sometimes the shoulders [2]. Symptoms can range from mild to severe in magnitude, and often involve pulling and jerking movements of the head and neck. Lateral head rotation and neck twisting are the symptoms most often observed in CD. Neck rigidity with pain may be experienced due to spasm and contraction of the involved muscles. CD is more common in women than in men, and is said to present in 5 to 20 per 100,000 individuals [3]. It can be of primary or secondary aetiology. Primary (idiopathic) dystonia occurs in the absence of an identifiable cause. There is no known structural abnormality in the central nervous system, or concomitant disease. Primary CD is associated with a hereditary component in approximately 12% of cases [4], and may be linked to previous neck injury [5]. Secondary dystonias are a consequence of disease processes, and have a clear cause which can be inherited or acquired. They are often linked to the use of certain medications (e.g. neuroleptics) or excessive toxin introduction into the body (e.g. in carbon monoxide poisoning), or to structural lesions primarily of the basal ganglia, such as trauma

ISSN 0953-4180/13/$27.50 © 2013 – IOS Press and the authors. All rights reserved or vascular insults. Dystonic symptoms may also manifest in neurodegenerative diseases such as Wilson's disease [6].

A number of medical therapies have been trialled for the treatment of CD. Botulinum toxin (BT), a neurotoxin produced by the bacterium *Clostridium botulinum*, causes impairment of neuromuscular transmission leading to flaccid paralysis [7]. It can be divided in to seven serotypes (BT A-G). Since the late 1980s, botulinum toxin type A (BTA) has been injected into symptomatic muscles to relieve the symptoms of CD, thus improving patient quality of life. However some patients do not respond well to BTA, or may develop resistance to it. Thus more recently botulinum toxin type B (BTB) injections have been used for CD treatment. For both serotypes of BT, different doses have been, and still are, being investigated as to which is the most efficacious in terms of maximal clinical effect and minimal adverse events. Prior to BT therapies, a variety of drug therapies were used for CD. Trihexyphenidyl, an anticholinergic, was the first line treatment for CD before the introduction of BTA to the market as it was the only drug deemed to be consistently efficacious [8].

This systematic review focuses on the available evidence on the safety and efficacy of a range of CD treatments. It aims to investigate which the most effective treatment is, based on the reviewed evidence-based literature.

## 2. Methods

A systematic review has been conducted in accordance to the Prisma guidelines (REF) to appraise existing literature regarding the effectiveness of different treatments used for CD. The studies were collected via the health sciences databases *Medline*, *PsycInfo*, *The Cochrane Library* and *PubMed*.

### 2.1. Search methods

Figure 1 shows a step-by-step approach to the searches performed on Medline with the respective number of results obtained. Similar searches were subsequently conducted on the other healthcare databases.

A simple, broad search was initially carried out to attain papers regarding the treatment of dystonias (i). This provided a vast number of hits, many of which were not relevant to the research topic, e.g. papers concerning different types of dystonia, or those mentioning dystonias or treatment in an unfitting context.

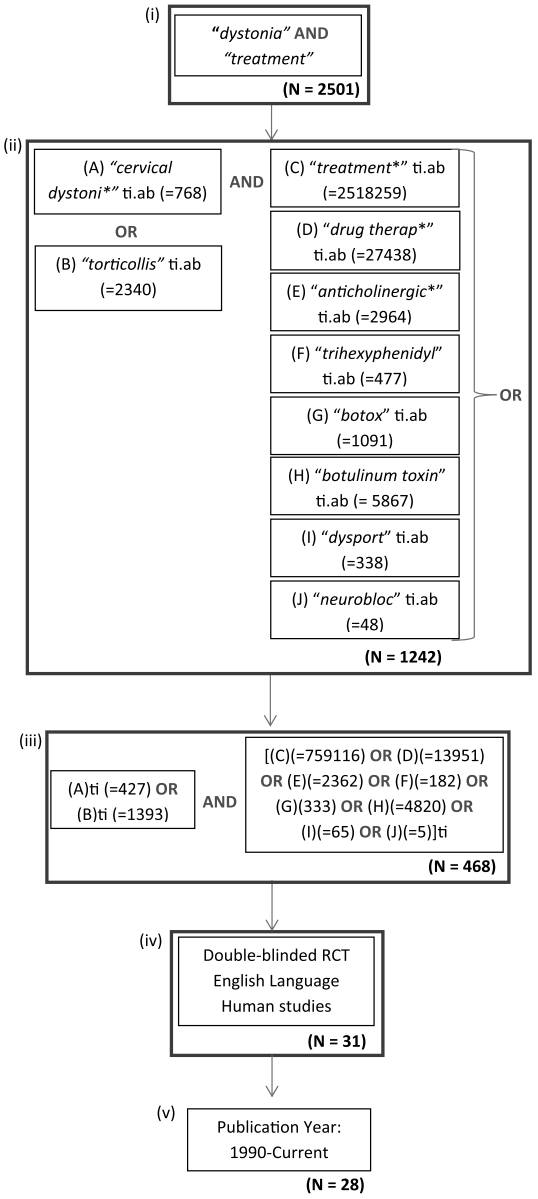

Fig. 1. Flow-chart detailing the literature search.

The search was therefore refined by using more precise terminology. "cervical dystoni*" was searched; 'cervical' to find papers more specific to the type of dystonia in question, and 'dystoni*' to include a number of terms such as 'dystonia', 'dystonias' and 'dystonic'. This was combined with "torticollis" as the two phrases are often interchanged. These terms were added to phrases related to the treatment of CD for example "treatment", "drug therap*" and specific treatment names such as "botulinum toxin". This search (ii) looked for those papers containing these expressions

Table 1
Placebo-controlled studies investigating the safety and efficacy of botulinum toxin type A in the treatment of cervical dystonia

| Ref | Author year | Country | Primary outcome measure | Sample size | BTA dose (units) | Main findings | Main limitations of study |
|---|---|---|---|---|---|---|---|
| [12] | Truong, Daniel, Brodsky et al. 2010 | USA | TWSTRS VAS | 116 | 500 | Significant decrease from baseline in mean TWSTRS scores and VAS pain scores and symptom assessments with tx. AEs mild-moderate as expected. | – VAS scales subjective to patient |
| [13] | Moore AP, Blumhardt LD. 1990 | UK | Tsui scale | 20 | 12.5 (ng) | Significant improvement in tx group. Main AE was dysphagia (dose-related). | – Tx group ($n = 16$) much larger than placebo group ($n = 4$)<br>– Small sample size |
| [14] | Lorentz IT, Subramaniam SS, Yiannikas C. 1991 | Australia | Tsui scale | 23 | 150 | Significant improvement with tx. Main AE pain at injection site. No serious or systemic AEs. | – Small sample size |
| [15] | Wissel J, Kanovsky P, Ruzicka E et al. 2001 | Austria | Tsui scale | 68 | 500 | Significant results showing more symptomatic improvement with tx than placebo. More AEs reported in tx group. | – Only patients with a Tsui score >9 (moderate-severe CD) included (study not representative of entire population with CD) |
| [16] | Poewe W, Deuschi G, Nebe A et al. 1998 | Germany | Tsui scale | 75 | 250, 500, 1,000 | Significant dose-response improvement with tx. Also dose-response relationship between tx and AEs. | – Dose-response relationship of AE's limits the maximum dose tested (even though higher doses correlate to improved efficacy) |

Abbreviations: TWSTRS, Toronto Western Spasmodic Torticollis Rating Scale; VAS, Visual Analogue Scale; tx, treatment; AE, adverse effect; THP, trihexyphenidyl.

within the title (ti) and abstract (ab) fields.

This greatly narrowed down the number of results obtained, but the majority were still not specifically related to the research topic. Many of the papers only briefly mentioned the key words in their abstracts. Therefore the same search was repeated but this time limited to those studies which included the key words in only their title fields (iii). This narrowed the number of results further, extracting more relevant literature to the research topic.

The search was then refined to involve only double-blinded, randomised controlled trials published in the English language. Only human studies were included (iv). Further modification of the search to consider more recent evidence was carried out by setting the historical limits from 1990 to present (v). This did not considerably affect the number of results, probably because of the increasing evidence related to CD therapies in more recent years, and the very little literature regarding it in the past.

Grey literature surrounding the research topic was also explored by reviewing the contents tables of pertinent journals, e.g. '*Neurology*' and '*Movement disorders*', and scanning the reference lists from relevant articles for further associated literature. 'Google' searches were also conducted to obtain literature which may not have been found via other search methods.

Only randomised, controlled, double-blinded trials published from 1990 to date were included in this review. All participants included in the reviewed studies had a diagnosis of idiopathic CD, and there was no restriction upon age or previous medication use for CD. When evaluating the efficacy of BT treatment, those studies using electromyography (EMG) assistance during treatment were manually excluded.

### 2.2. Outcome measures

A number of scales are used to score the severity of CD. This review includes those with the primary outcome measure of the Tsui scale or TWSTRS (Toronto

Table 2
Placebo-controlled trials investigating the safety and efficacy of botulinum toxin type B in the treatment of cervical dystonia

| Ref | Author year | Country | Main outcome measures | Sample size | BTB dose (units) | Main findings | Main limitations of study |
|---|---|---|---|---|---|---|---|
| [17] | Brashear, Allison, Lew et al. 1999 | USA | TWSTRS | 109 | 5,000, 10,000 | SS results showing BTB to be safe and efficacious for patients with BTA-responsive CD. A higher dose correlates to a better clinical effect. | – Four patients withdrew from study<br>– Short time-scale<br>– One treatment cycle |
| [18] | Brin, Mitchell F, Lew et al. 1999 | USA | TWSTRS | 77 | 10,000 | SS results showing BTB to be safe and efficacious for patients with BTA-resistant CD despite AE's (dry mouth and dysphagia) | – Short time-scale<br>– One treatment cycle |
| [19] | Lew MF, Adornato BT, Duane DD et al. 1997 | USA | TWSTRS | 122 | 2,500, 5,000, 10,000 | BTB is safe and efficacious for patients with CD. SS dose-response relationship between BTB and clinical effect. | – Short time-scale<br>– One treatment cycle |

Table 3
Studies comparing the use of botulinum toxin type A and botulinum toxin type B in the treatment of cervical dystonia

| Ref | Author year | Country | Main outcome measures | Sample size | Main findings | Main limitations of study |
|---|---|---|---|---|---|---|
| [20] | Pappert EJ, Germanson T et al. 2008 | USA | TWSTRS | 111 | SS results showing BTA and BTB to have similar clinical effectiveness in toxin-naïve patients. BTB is more likely to cause mild dry mouth. | – Only one treatment dose investigated per BT serotype |
| [21] | Comella CL, Jankovic J, Shannon KM et al. 2005 | USA | TWSTRS | 139 | SS results showing BTA and BTB to be effective in the tx of CD. BTA has longer duration of clinical action and a lower side effect profile than BTB. | – Only one treatment dose investigated per BT serotype |

Western Spasmodic Torticollis Rating Scale). This is to enable objective analysis and comparison of the results of the studies.

TWSTRS takes into account severity, disability and pain associated with CD. Each of these is scored independently and totalled to form a score in between 0 and 87, being the best and worst scores respectively [9]. The Tsui scale considers the degree of postural deviation, the presence of head tremor and the type of movement (intermittent or continuous) in CD [10]. This scale is also numerically scored (from 0 to 25). Evidence shows there to be a significant correlation between a reduced score in both scales post-BT treatment [11].

Secondary outcome measures did not play a role in the determination of which studies are included in this review.

## 3. Results

Tables 1–3 show the results of the systematic literature review on the treatment of CD.

The studies in Table 1 [12–16] all provide statistically significant results demonstrating BTA injections to be safe and efficacious for the treatment of CD when compared to placebo.

Study [12] operated multiple treatment cycles over a fairly long time period, thus showing BTA injections to have a good long-term safety profile. This was most significant by week 4 post-administration, with effects lasting until week 12.

Despite small sample sizes, Moore et al. [13] and Lorentz et al. [14] show statistically significant results. Study [13] shows the main adverse effect of BTA treatment to be dysphagia. All patients continued treatment regardless of adverse effects due to perceived net benefit; the belief that the symptomatic relief from CD, e.g. neck weakness, outweigh the side effects of treatment. Study [14] shows the main side effect to be pain at the injection site, in the absence of systemic or serious adverse effects of BTA.

Even though considerable response to BTA treatment was demonstrated in study [15], Wissel et al. show CD patients to also clinically respond to placebo. This

Table 4
Study comparing the use of trihexyphenidyl (anticholinergic) and botulinum toxin type A in the treatment of cervical dystonia

| Ref. | Author Year | Country | Main outcome measures | Sample size | Main findings | Main limitations of study |
|---|---|---|---|---|---|---|
| [22] | Brans JW, Lindeboom R, Snoek JW et al. 1996 | The Netherlands | TWSTRS, Tsui scale, General Health Perception Scale | 66 | SS results show that BTA is more effective than THP in CD treatment, with a lower AE profile. | – General health perception scale is subjective<br>– Small sample size<br>– Short duration of study<br>– No other anticholinergic drugs investigated<br>– THP only compared to BTA (no other treatments)<br>– Dosing error in five patients<br>– Two patients withdrew from study |

improvement was not in the Tsui score, but in subjective secondary outcome scales self-reported by the patients and assessors.

When investigating the optimal dose of BTA in the treatment of CD, Poewe et al. [16] found that higher doses positively correlate with clinical effect and adverse effects. A statistically significant clinical improvement was noted in patients treated with 500 or 1,000 units of BTA. The improvement in the 250 unit treatment group was not significant. Therefore it was concluded that patients should initially be treated with milder doses of BTA, which can be titrated up depending on the efficacy of that dose and how well the adverse effects of higher doses are tolerated by the patient.

Brashear et al. [17] investigated the effects of BTB in BTA-responsive patients, and [18] in those who are BTA-resistant. Both studies provide statistically significant results showing that BTB is safe and efficacious in treating CD. However Brin et al suggest that the use of BTB is more effective in those patients who are BTA-resistant.

Lew et al. [19] investigated the effects of BTB upon BTA-responsive and -resistant CD patients. By utilising three different doses of BTB, this study effectively shows that a spectrum of doses is clinically safe and efficacious for the treatment of CD. It is also indicates a statistically significant dose-response relationship to treatment.

Thus evidence leads us to believe that BTB is a safe and effective treatment for CD in patients regardless of their response to BTA, with its clinical effect positively correlating to treatment dose.

Few studies compare BTA and BTB to each other; however those which do conclude that they have a similar clinical effect. Pappert et al. [20] did not find any significant difference in duration of treatment effect (13.1 weeks (BTA) vs 13.7 weeks (BTB)), or adverse effects (dysphagia and pain at injection site) in toxin-naïve patients. Mild dry mouth was higher for BTB, but there was no significant difference between the groups in the frequency of moderate or severe dry mouth.

Comella et al. [21] compare the two BT serotypes in BTA-responsive patients. The clinical improvement was similar between the different serotypes of BT at 4 weeks, but those treated with BTA had a significantly longer duration of action than those with BTB (14 weeks vs 12.1 weeks respectively). The adverse effects of dysphagia and dry mouth were significantly higher in those treated with BTB.

Finally, Brans et al. compared the efficacy of trihexyphenidyl to the efficacy of BTA [22]. The evidence suggests that BTA is more effective a treatment than trihexyphenidyl for CD, with less adverse effects.

## 4. Discussion

The main objectives of this review are to observe and compare the efficacy and safety of BTA, BTB and trihexyphenidyl in the treatment of CD.

The evidence from Table 1 concludes that BTA is a safe and efficacious treatment for CD, with anticipated mild to moderate adverse effects. Table 2 shows similar results regarding the treatment of CD with BTB. When compared to one another (Table 3), the two serotypes of BT are shown to have a similar clinical benefits, but studies disagree on their respective duration of action; one study claims that they are effective for a similar period of time and the other argues that the effects of BTA injections last longer. However both studies show a higher number of adverse effects associated with

BTB. On comparison of trihexyphenidyl treatment to BTA it is established that BTA has a superior clinical effect and lower side effect profile, making it the more efficacious treatment option of the two.

### *4.1. Limitations*

The studies included in this review are double-blinded, randomised controlled trials. This reduces measurement bias and causality, and keeps the type of evidence analysed uniform as only primary research is used. However there are also limitations to this type of study. Randomised controlled trials are often associated with ethical concerns; if treatment A is *believed* to be superior to treatment B in certain patients, it can be seen as unethical to potentially allocate the less efficacious or less well understood treatment to those patients. However without trials there will be no way to determine whether or not these treatments are more, less or equally efficacious as those with more evidence surrounding their uses. Therefore the chance of fabrication cannot completely be ruled out.

The primary outcome measures used for this review are limited to the TWSTRS and Tsui scales, reducing measurement bias. However the secondary outcome scales were not considered. The majority of these were subjective to the patient or to the investigators' personal opinions, thus being of limited benefit when comparing groups of patients. Study [15] demonstrates a flaw in using multiple scales, including subjective scales, to analyse the results of a number of studies: it shows CD patients to have had a considerable response to placebo when trialled against BTA. Most of the patients in the placebo group did not improve in terms of Tsui score, but had 'improved' when questioned subjectively, or assessed by the blinded investigators. Thus it is difficult to deem them a reliable source of information. Nonetheless they may be useful in measuring improvement or decline in an individual patient's symptoms. Some studies used the subjective VAS scale [12] or General Health Perception Scale [22] as part of their *primary* outcome measures, but this was alongside the Tsui scale or TWSTRS so were not excluded from this review.

The literature surrounding the treatment of CD is sparse, possibly because CD itself is relatively rare and BT has been the first line treatment for some time now. As only trials conducted after 1990 have been included, markedly few studies on the efficacy of trihexyphenidyl and other drugs were found.

Furthermore a number of the studies used small sample sizes, limiting the dependability of the results. However the majority of results obtained were statistically significant. In this review, results from studies are only considered statistically significant if within the conventional levels of significance ($p < 0.05$).

The duration of some studies also limited their reliability. For example, study [22] was conducted over a fairly short time scale. Trihexyphenidyl is associated with a maximal clinical effect many weeks after use, and this may not have been observed in the somewhat short study. Also, the BTA injections were administered twice in eight weeks, a narrower clinical interval than in which it usually produces optimal effects. Furthermore, there was no restriction as to other interventions that the patients may be receiving during the trials. This may have affected the results (co-intervention bias). There was also potential for withdrawal bias: patients dropped out from some of the studies for a range of reasons, including no perceived benefit from treatment or placebo, and spontaneous remission.

When analysing studies regarding the effectiveness of BTA, some mentioned the use of 'Botox' and others 'Dysport'. These were not investigated separately, but were placed in the subgroup 'BTA'. The different formulations may have produced different results. Moreover, study [13] shows the adverse effects experienced with BTA to be dose-related, thus limiting the doses used in treatment (despite higher doses being positively correlated to clinical improvement in CD). This may have affected the results further, and prevented the comparison of a larger range of doses of BTA for CD treatment.

The studies discussing the effectiveness of BTB also have limitations as they only involve one treatment cycle over a relatively short period of time; thus these aren't useful in determining the long-term safety and efficacy of multiple treatments. Furthermore, in the studies comparing the efficacy of BTA and BTB, only one dose of each serotype of BT is investigated. Previous evidence shows that the adverse effects of BT in the treatment of CD are dose-related. Thus it is not necessarily appropriate to compare the adverse reactions to treatment unless a range of doses are experimented with.

The study by Brans et al. [22] is also limited as it compares trihexyphenidyl to only BTA treatment. Therefore the anticholinergic's effectiveness in comparison to other therapies is unknown. There was also a dosing error in this study, leading to unreliability of some of the results.

### *4.2. Suggestions for future research*

There are a number of ways in which this review can be improved or modified if repeated. Additional drug therapies (besides trihexyphenidyl and BT) for the treatment of CD could be explored, including other anticholinergic drugs. This may require extending the historical limits to before 1990. Studies carried out over a larger time scale could also be included to investigate the long-term effects of these treatments. Moreover only those studies with larger sample sizes could be explored, increasing the reliability of the outcomes. Secondary outcome scales could also be controlled and compared. Even though many of these are subjective and so not always completely reliable, they can be a useful tool for analysis.

If looking at a particular treatment for CD, it would be useful to investigate more studies related to optimal dosing. Or, if investigating treatment efficacy, the literature involving only one dose of that therapy should be included in the review to assure consistency during analysis. Additionally, in order to eliminate co-intervention bias, the studies compared in the review should include patients who are not/have not previously received any treatment for CD. This would further ensure uniformity between studies.

Finally, the efficacy studies for BTA and BTB were narrowed to those not involving EMG guidance. In future, studies using EMG could be reviewed and compared to those which do not use it to see if it is an effective tool in the monitoring and treatment of CD.

## 5. Conclusions

This systematic review investigates the safety and effectiveness of different treatments for CD, and explores which is the most suitable in terms of maximal clinical benefit and minimal adverse events.

The results reveal that all of the therapies investigated (BTA, BTB and trihexyphenidyl) are both safe and efficacious treatments for CD. However, evidence reveals BT therapies to be more reliable for symptomatic relief and have fewer adverse effects than trihexyphenidyl. When comparing BTA to BTB, both were found to have a similar clinical benefit, with BTA possibly having a longer duration of action and a marginally better side effect profile.

Therefore, of the treatments investigated it can be concluded that BTA is probably the best treatment of choice for CD. BTB is also safe and likely to be just as efficacious a treatment in those patients who are unresponsive or have become resistant to BTA.

Future studies may include the investigation of other treatment options for CD, and possibly larger studies conducted over an extended time-period to gain more reliable results which look at the long-term implications of CD therapies. It may also be useful to look at the Cochrane literature reviews regarding the pharmacological treatment of CD, and compare the findings in them to those acquired in this review.
